# Supplementary material for: Antitumor Activity of an Anti-EGFR/HER2 Bispecific Antibody in a Mouse Xenograft Model of Canine Osteosarcoma
Source: Pharmaceutics. 2022 Nov 17;14(11):2494. doi: 10.3390/pharmaceutics14112494 (PMC9697293; doi:10.3390/pharmaceutics14112494)
Supplement: Supplementary file 1 [file pharmaceutics-14-02494-s001.zip › pharmaceutics-1967426-supplementary.pdf]

Supplementary Figures

Antitumor Activity of an Anti-EGFR/HER2 Bispecific Antibody in a Mouse Xenograft Model of Canine Osteosarcoma

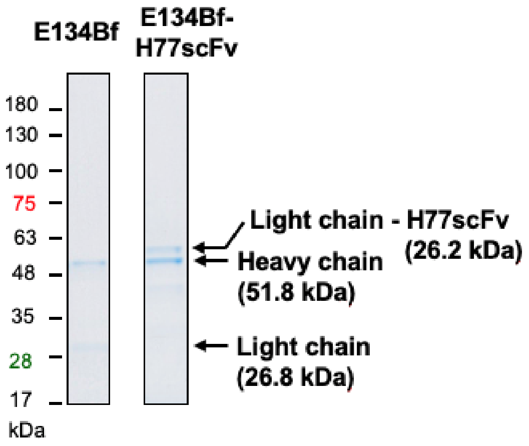

Supplementary Figure S1. Purity of E134Bf-H77scFv. E134Bf (0.5 µg) and E134Bf-H77scFv (0.5 µg) were denatured by sodium dodecyl sulfate (SDS) sample buffer (Nacalai Tesque, Inc.). Proteins were separated on 5%–20% polyacrylamide gel (FUJIFILM Wako). The gel was stained by Bio-Safe CBB G-250 Stain (Bio-Rad Laboratories, Inc.).

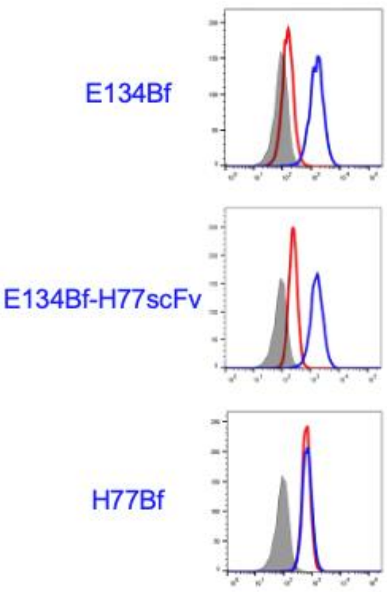

Supplementary Figure S2. Flow cytometry using the EGFR peptide containing E134Bf epitope. E134Bf (1 µg/ml), E134Bf-H77scFv (1 µg/ml), and each mAb plus the EGFR peptide (aa 375-394, 30 µg/ml) [44], or control (blocking buffer) were reacted with D-17 cells for 30 min at 4℃, followed by treatment with Alexa Fluor 488-conjugated anti-dog IgG. Fluorescence data were analyzed using the SA3800 Cell Analyzer (Sony Corp.). Blue lines; each mAb, red lines; each mAb + EGFR peptide (aa 375-394), filled gray; control.
